# New Cerebroside and Nucleoside Derivatives from a Red Sea Strain of the Marine Cyanobacterium *Moorea producens*

**DOI:** 10.3390/molecules21030324

**Published:** 2016-03-09

**Authors:** Diaa T.A. Youssef, Sabrin R.M. Ibrahim, Lamiaa A. Shaala, Gamal A. Mohamed, Zainy M. Banjar

**Affiliations:** 1 Department of Natural Products, Faculty of Pharmacy, King Abdulaziz University, Jeddah 21589, Saudi Arabia; gamals2001@yahoo.com; 2 Department of Pharmacognosy and Pharmaceutical Chemistry, College of Pharmacy, Taibah University, Al Madinah Al Munawwarah 30078, Saudi Arabia; sribrahim@taibahu.edu.sa; 3 Department of Pharmacognosy, Faculty of Pharmacy, Assiut University, Assiut 71526, Egypt; 4 Natural Products Unit, King Fahd Medical Research Center, King Abdulaziz University, Jeddah 21589, Saudi Arabia; lshalla@kau.edu.sa; 5 Suez Canal University Hospital, Suez Canal University, Ismailia 41522, Egypt; 6 Department of Pharmacognosy, Faculty of Pharmacy, Al-Azhar University, Assiut Branch, Assiut 71524, Egypt; 7 Department of Clinical Biochemistry, Faculty of Medicine, King Abdulaziz University, Jeddah 21589, Saudi Arabia; zmbanjar@yahoo.com

**Keywords:** marine cyanobacterium, *Moorea producens*, cerebroside, nucleosides, cytotoxic activity

## Abstract

In the course of our ongoing efforts to identify marine-derived bioactive compounds, the marine cyanobacterium *Moorea producens* was investigated. The organic extract of the Red Sea cyanobacterium afforded one new cerebroside, mooreaside A (**1**), two new nucleoside derivatives, 3-acetyl-2′-deoxyuridine (**2**) and 3-phenylethyl-2′-deoxyuridine (**3**), along with the previously reported compounds thymidine (**4**) and 2,3-dihydroxypropyl heptacosanoate (**5**). The structures of the compounds were determined by different spectroscopic studies (UV, IR, 1D, 2D NMR, and HRESIMS), as well as comparison with the literature data. Compounds **1**–**5** showed variable cytotoxic activity against three cancer cell lines.

## 1. Introduction

Nucleosides and cerebrosides are found in both terrestrial and marine organisms. Cerebrosides are composed of a hydrophobic part named ceramide, which is linked to one sugar moiety [1]. Cerebrosides play an important role in major cellular processes including growth, morphogenesis and cell differentiation. They also affect cell signaling by controlling the assembly and specific activities of plasma membrane proteins [2,3]. Nucleosides are derivatives of glycosylamines, which are central metabolites in all life forms [4]. Nucleotides, the building block of DNA and RNA are composed mainly of nucleosides with at least one phosphate group. Nucleoside-derived compounds are used effectively in treatment of tumors, viral infections and malignant neoplasms [5,6]. 

Marine cyanobacteria are vital producers of diverse chemical entities with significant bioactivities [7,8,9,10]. The genus *Moorea* (formerly *Lygnbya*) [11] has been proven to be a rich source for novel bioactive compounds of different classes [11,12]. Cyanobacteria derived compounds display a wide range of biological activities including those that are antimicrobial, antiproliferative, anticancer, antifeedant, antifungal, and anti-inflammatory [13,14,15,16,17]. Previous work on the Red Sea cyanobactrium *Moorea producens* revealed the presence of nitrogen-containing compounds, polyketides and peptides [16,17]. In continuation of our ongoing interest to allocate new bioactive compounds from Red Sea marine cyanobacteria [16,17,18,19], we here focus on the Red Sea strain of the cyanobacterium *Moorea producens*. In this paper, we reported the isolation and structure determination of a new cerebroside, mooreaside A (**1**), two new nucleoside derivatives, 3-acetyl-2′-deoxyuridine (**2**) and 3-phenylethyl-2′-deoxyuridine (**3**), along with the known compounds thymidine (**4**) and 2,3-dihydroxypropyl heptacosanoate (**5**) from the organic extract of the marine cyanobacterium *Moorea producens*. The structures of the compounds were determined using different spectroscopic techniques. The cytotoxic activity of the compounds against three cancer cell lines will be discussed. 

## 2. Results and Discussion

### 2.1. Purification of Compounds ***1**–**5***

**Figure 1:**
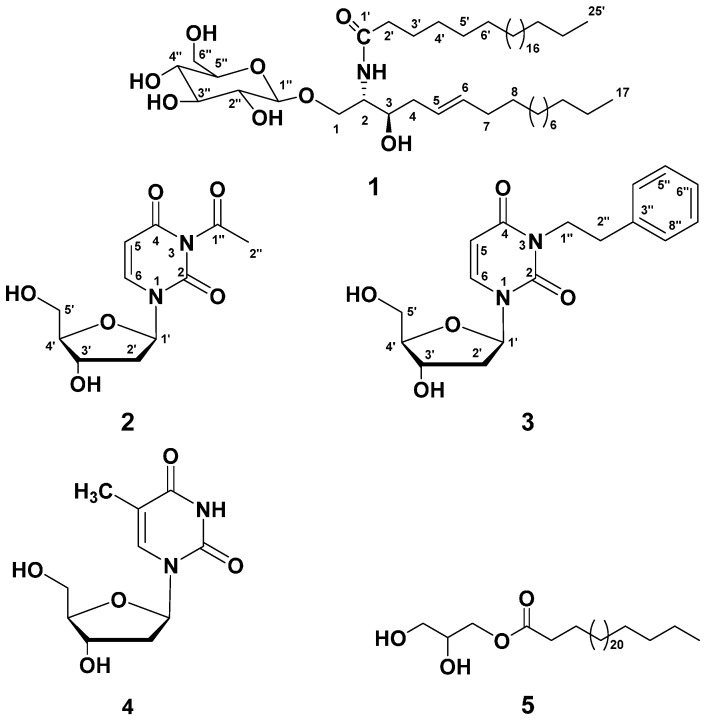
Structures of compounds **1**–**5**.

Samples of *M*. *producens* were extracted with a mixture of MeOH/CH_2_Cl_2_ (2:1). The organic extract was subjected to chromatographic separation on normal SiO_2_, Sephadex LH-20, and RP-18 columns to provide three new compounds **1**–**3** and two known compounds **4** and **5** (Figure 1). The isolated compounds were evaluated for their cytotoxic activity. 

### 2.2. Structure Elucidation of Compound ***1***

**Figure 2:**
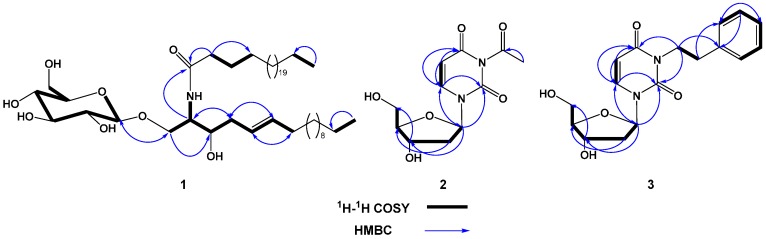
Key ^1^H-^1^H COSY and HMBC correlations of compounds **1**–**3**.

**Figure 3:**
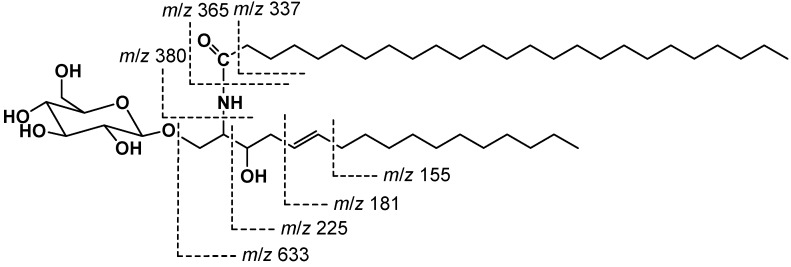
Key MS fragments of **1**.

Compound **1** (Figure 1) was obtained as a colorless amorphous powder. Its molecular formula was suggested as C_48_H_93_NO_8_ on the basis of the HRESIMS quasi-molecular ion peak at *m*/*z* 812.6982 [M + H]^+^ and ^1^H- (Figures S1–S3) and ^13^C-NMR (Figures S4 and S5) spectral analyses, requiring three degrees of unsaturation. Its IR spectrum showed characteristic absorption bands at 3435 (hydroxyl), 3320 and 1635 (amide), 3005 and 960 (olefinic), and 1150 (C–O) cm^−1^, suggesting the cerebroside nature of **1** [20,21,22,23]. The ^1^H-NMR spectrum of **1** showed two signals at δ_H_ 5.35 (dt, *J* = 15.3, 7.6 Hz, H-5) and 5.37 (dt, *J* = 15.3, 7.1 Hz, H-6) in ^1^H-NMR spectrum characteristic for the presence of a *di*-substituted olefinic moiety (Table 1) which was supported y COSY correlation (Figure S6). These protons correlated to the carbon signals at δ_C_ 129.9 and 128.8, respectively, in the HSQC spectrum (Figure S7). The *trans* (*E*) configuration of the double bond was proven by the large vicinal coupling constant value (*J*_5,6_ = 15.3 Hz) and the chemical shifts of the carbons next to the double bond at δ_H_ 32.2 (C-4) and 32.0 (C-7) [24,25,26]. The location of the olefinic moiety at C-6/C-7 was established based on the HMBC cross peaks from H-5 to C-3, C-4, and C-7 and from H-6 to C-4, C-5, and C-7 (Figure S8). In the ^1^H, ^13^C, and multiplicity-edited HSQC spectra, the signals at δ_H_ 4.28 (d, *J* = 7.7 Hz, H-1′′)/δ_C_ 104.0 (C-1′′) revealed the presence of a β-glucopyranoside moiety. This was also confirmed by the ESIMS fragment ion peak at *m*/*z* 633 [MH − Glc]^+^. The attachment of the glucose moiety at C-1 was established by the downfield shift of C-1 (δ_C_ 68.5) and HMBC correlation of H-1′′ (δ_H_ 4.28) with C-1. Moreover, the ^1^H-NMR spectrum of **1** showed signals at δ_H_ 3.61 (m, H-3), 3.97 (m, H-2), 3.91 and 3.74 (each m, H-1) attributable to oxymethine, N*H*-bonded methine, and oxymethylene groups, respectively. They correlated with the carbon signals at 73.4 (C-3), 59.3 (C-2), and 68.5 (C-1) in the HSQC spectrum. ^1^H-^1^H COSY cross peaks were observed between the N*H* proton (δ_H_ 7.51) and H-2, which coupled to H-1 and H-3, suggesting the presence of hydroxyl group in the long chain base. This was further confirmed by the HMBC correlations of H-1 to C-2 and C-3, H-2 to C-1, C-3, and C-4, H-3 to C-1 and C-2, and 2-N*H* to C-2 and C-3 (Figure 2). Comparing ^13^C-NMR spectrum of **1** with those of glucosyl-*erythro*-ceramide and glucosyl-*threo*-ceramide, proved the *erythro* configuration at C-2 and C-3 in the sphingosine part of **1** [27,28,29,30,31,32]. The length of the fatty acid chain (C-1′→C-25′) and base chain (C-1→C-17) was determined by the ESIMS. The EIMS spectrum of **1** showed characteristic fragment ion peaks at *m*/*z* 380 [CH_3_(CH_2_)_23_CONH]^+^, 365 [CH_3_(CH_2_)_23_CO]^+^, 337 [CH_3_(CH_2_)_23_]^+^, 225 [CH_3_(CH_2_)_10_-CH=CH-CH_2_(CHOH)]^+^, 181 [CH_3_(CH_2_)_10_-CH=CH]^+^, and 155 [CH_3_(CH_2_)_10_]^+^ (Figure 3), supporting the chain lengths of **1**. Methanolysis of **1** gave long chain base (LCB) and fatty methyl ester (FAME). The FAME in the *n*-hexane layer was identified as pentacosanoic acid methyl ester based on the GCMS molecular ion peak at *m*/*z* 396 [M]^+^. The LCB showed an EIMS molecular ion peak at *m*/*z* 285 [M]^+^, corresponding to (2*S*,3*R*,*E*)-2-aminoheptadec-5-ene-1,3-diol. Based on the above evidence and discussion, compound **1** was assigned as *N*-((2*S*,3*R*,5*E*)-3-hydroxy-1-*O*-((β-d-glucopyranosyl)heptadec-5-en-2-yl)pentacosanamide. Compound **1** was generically named mooreaside A.

### 2.3. Structure Elucidation of Compound ***2***

Compound **2** (Figure 1) was obtained as a white powder. Its HRESIMS gave a quasi-molecular ion peak at *m*/*z* 271.0927 [M + H]^+^, consistent with a molecular formula C_11_H_14_N_2_O_6_, requiring six degrees of unsaturation. The IR spectrum of **2** showed absorption bands at 3394 (hydroxyl) and 1693 (amide carbonyl) cm^−1^. These data in conjunction with characteristic UV absorption bands at λ_max_ 253 and 268 nm along with 1H NMR spectrum (Figures S9–S11) suggested the presence of uracil moiety in **2**. The ^13^C (Figure S12) and multiplicity-edited HSQC (Figure S13) spectra of **2** showed signals for 11 carbons including one methyls, two methylenes, five methines, and three quaternary carbonyls at δ_C_ 151.7 (C-2), 162.8 (C-4), and 183.4 (C-8). The ^1^H-^1^H COSY spectrum (Table 2) showed two *ortho*-coupled protons at δ_H_ 5.69 (d, *J* = 8.5 Hz, H-5) and 7.98 (d, *J* = 8.5 Hz, H-6). These protons correlated to the carbon signals at δ_C_ 102.6 and 142.5, respectively in the HSQC, indicating the presence of uracil moiety in **2**. This was confirmed by the HMBC (Figure S14) cross peaks from H-5 to C-4 and C-6 and from H-6 to C-2 and C-4. Moreover, the ^1^H- and ^13^C-NMR signals at δ_H_ 6.27 (t, *J* = 6.8 Hz, H-1′)/δ_C_ 86.6 (C-1′), 2.28 (m, H_2_-2′)/41.4 (C-1′), 4.38 (m, H-3′)/72.2 (C-3′), 3.89 (m, H-4′)/89.0 (C-4′), and 3.75 (m, H-5′a) and 3.71 (m, H-5′b)/62.8 (C-5′) supported the presence of 2′-deoxyribose moiety [33] in **2**. The connectivity of this moiety at *N*-1 of the uracil moiety was established by the HMBC cross peaks from H-6 to C-1′ and from H-1′ to C-2 and C-6. Furthermore, signals for an acetyl group at δ_H_ 1.95 (3H, s, H-2′′)/δ_C_ 23.5 (C-2′′) and 183.4 (qC, C-1′′) were observed [34]. This was confirmed by the ESIMS fragment ion peak at *m*/*z* 228 [MH – COCH_3_]^+^. Based on the ^13^C chemical shifts, the acetyl group was assigned at N-3 of the uracil moiety, completing the molecular formula of **2** and the degrees of unsaturation. Consequently, **2** was assigned as 3-acetyl-2′-deoxyuridine and is reported here as a new natural product.

### 2.4. Structure Elucidation of Compound ***3***

Compound **3** (Figure 1) was obtained as white amorphous powder with a molecular formula C_17_H_20_N_2_O_5_ as determined from its HRESIMS quasi-molecular ion peak at *m*/*z* 333.1446 [M + H]^+^, requiring nine degrees of unsaturation. The 1D and 2D-NMR (Figures S15–S20) spectral data (Table 2) of **3** were quite similar to those of **2** except the absence of the signals associated with acetyl group at *N*-3. Instead, new signals at δ_H_/δ_C_ 7.35 (2H, d, *J* = 8.5 Hz, H-5′′,7′′)/130.0 (C-5′′,7′′), 7.27 (2H, brd, *J* = 8.5 Hz, H-4′′,8′′)/129.8 (C-4′′,8′′), 7.25 (1H, t, *J* = 8.5 Hz, H-6′′)/128.3 (C-6′′), 2.95 (2H, t, *J* = 7.6 Hz, H-2′′)/34.7 (C-2′′), and 3.16 (2H, t, *J* = 7.6 Hz, H-1′′)/34.7 (C-1′′) were observed. The signals suggested the presence of a *N*-bonded phenylethyl moiety. This was established by ^1^H-^1^H COSY cross peaks, and further confirmed by HMBC correlations (Figure 2) from H-2′′ to C-4′′/C-8′′ and C-1′′, and from H-1′′ to C-2′′ and C-3′′. The HMBC cross peaks of H-1′′/C-2 and H-1′′/C-4 supported the placement of phenylethyl moiety at *N*-3. Moreover, the ESIMS of **2** gave a characteristic fragment ion peak at *m*/*z* 228 [MH − CH_2_CH_2_C_6_H_5_]^+^, indicating the loss of a phenylethyl moiety [35]. Thus, compound **3** was assigned as 3-phenylethyl-2′-deoxyuridine and is considered a new natural product.

The known compounds were identified as thymidine (**4**) [36] and 2,3-dihydroxypropyl heptacosanoate (**5**) [37,38] by analysis of their spectroscopic data and comparison with those in the literature.

The compounds were evaluated for their cytotoxic activities against three cancer cell lines, including colorectal carcinoma (HCT-116, ATCC CCL-247), hepatocellular carcinoma (HepG2, ATCC HB-8065), and breast cancer (MCF-7, ATCC HTB-22). Compounds **1**–**3** showed moderate activity towards MCF-7 cancer cell line. Meanwhile, they were inactive towards HCT-116 and HepG2 cancer cell lines (Table 3).

## 3. Experimental Section 

### 3.1. General Experimental Procedures 

Optical rotations were measured on a JASCO DIP-370 digital polarimeter (Jasco Co., Tokyo, Japan) at 25 °C at the sodium D line (589 nm). UV spectra were recorded on a Hitachi 300 spectrometer (Hitachi High-Technologies Corporation, Kyoto, Japan). The IR spectra were acquired with a Shimadzu Infrared-400 spectrophotometer (Shimadzu, Kyoto, Japan). EIMS was recorded on a JEOL the mass route JMS.600H mass spectrometer **(**JEOL USA, Inc., Peabody, MA, USA). HRESIMS spectra were performed on a Micromass Qtof 2 mass spectrometer (Bruker, Rheinstetten, Germany). GCMS analysis was performed on GCMS Hewlett-Packard 5890 GC (Hewlett-Packard, Wilmington, DE, USA) equipped with a mass-selective detector MSD 5970 MS, a split injector and a fused-silica HP-5 column (25 m × 0.2 mm; i.d. 0.33 mm film); column temp. 230 °C, carrier N_2_, flow rate 30 mL/min. NMR spectra were determined on Bruker Ascend™ 850 (850 MHz) (Bruker BioSpin, Billerica, MA, USA) using CD_3_OD and CDCl_3_ as solvent. The HPLC separation was performed on a RP-18, 250 × 10 mm, 5 μm Phenomenex Luna column using H_2_O/ACN as mobile phase, detected at 220 nm with a flow rate of 2.0 mL/min. Column chromatographic separations were performed on SiO_2_ 60 (0.04–0.063 mm, Merck, Darmstadt, Germany), Sephadex LH-20 (0.25–0.1 mm, Merck), and RP-18 (0.04–0.063 mm, Merck). Pre-coated SiO_2_ 60 F_254_ plates (Merck) were used for TLC. Compounds were detected by UV absorption at λ_max_ 255 and 366 nm followed by spraying with *p*-anisaldehyde/H_2_SO_4_ reagent and heating at 110 °C for 1–2 min.

### 3.2. Biological Materials

The marine cyanobacterium *Moorea producens* was collected from the Red Sea by hand at 1 m depth near Jeddah, Saudi Arabia. The cyanobacterium was identified by Dr. Ali Gab-Alla, Faculty of Science of Suez Canal University. A voucher sample was kept at Department of Natural Products and Alternative Medicine, Faculty of Pharmacy, King Abdulaziz University under the registration code No. 2013-LM5.

### 3.3. Extraction and Purifications of Compounds ***1**–**5***

The freeze-dried cyanobacterium *M. producens* (35 g) was extracted at room temperature with a mixture of MeOH/CH_2_Cl_2_ (2:1, 1 L × 4). The combined extracts were evaporated under reduced pressure to give a greenish organic extract. The extract (980 mg) was subjected to flash SiO_2_ column using *n*-hexane/EtOAc/MeOH gradients to give 7 fractions (F1–F7). Fraction F2 (*n*-hexane/EtOAc 8:2, 65 mg) was chromatographed over SiO_2_ column (35 g × 50 cm × 2 cm) using *n*-hexane/EtOAc (97:3 to 90:10) to give impure **5**, which was purified by C18 semi-preparative HPLC column using 30% ACN to give **5** (5.3 mg). Fraction F4 (55 mg, EtOAc) was chromatographed over SiO_2_ column (30 g × 50 cm × 2 cm) using CHCl_3_/MeOH (95:5 to 85:15) elution afforded impure **1**, which further purified on C18 HPLC semi-preparative column using 55% ACN to give pure **1** (9.6 mg). Sephadex LH-20 column chromatography (50 g × 50 cm × 3 cm) of fraction F6 (68 mg) using MeOH as solvent system gave impure **2** and **3**. Final purification of the two compounds was achieved on RP-18 column (60 g × 50 cm × 3 cm) using MeOH/H_2_O (50:50 to 90:10) elution to give **2** (4.3 mg) and **3** (3.2 mg). HPLC purification of F7 (35 mg) on C18 HPLC semi-preparative column using 60% ACN gave **4** (3.5 mg). 

Mooreaside A (**1**): Colorless amorphous powder; ${\left[\alpha \right]}_{D}^{25}$ + 4.8 (*c* 0.2, MeOH); IR (film) ν_max_ 3435, 3320, 3005, 1635, 1150, 960 cm^−1^; HRESIMS *m*/*z* 728.6031 (calcd for C_42_H_82_NO_8_, 728.6040 [M + H]^+^); NMR spectral data, see Table 1.

3-Acetyl-2′-deoxyuridine (**2**): White powder; ${\left[\alpha \right]}_{D}^{25}$ + 18.6 (*c* 1.0, MeOH); UV (MeOH) λ_max_ (log ε) 253 (2.36), 268 (2.89) nm; IR (film) ν_max_ 3294, 2956, 1693 cm^−1^; HRESIMS *m*/*z* 271.0927 [M + H]^+^ (calcd for C_11_H_15_N_2_O_6_, 271.0930 [M + H]^+^); NMR spectral data, see Table 2.

3-Phenylethyl-2′-deoxyuridine (**3**): White amorphous powder; ${\left[\alpha \right]}_{D}^{25}$ + 14.1 (*c* 0.2, MeOH); UV (MeOH) λ_max_ (log ε) 257 (2.42), 269 (2.87) nm; IR (film) ν_max_ 3289, 2959, 1694, 970, 746 cm^−1^; HRESIMS *m*/*z* 333.1446 [M + H]^+^ (calcd C_17_H_21_N_2_O_5_, 333.1450); NMR spectral data, see Table 2.

### 3.4. Evaluation of the Cytotoxicity of the Compounds

The isolated compounds (**1**–**5**) were evaluated for their cytotoxic activity against colorectal carcinoma (HCT-116), hepatocellular carcinoma (HepG2), and breast cancer (MCF-7). The cells were obtained commercially from ATCC. The cytotoxicity was evaluated by the sulforhodamine B (SRB) assay, as described previously [39]. Doxorubicin was used as positive control drug (Table 3).

### 3.5. Methanolysis

Compound **1** (4.5 mg) was treated with 6 mL of 1N HCl in MeOH at 90 °C for 15 h in a sealed ampoule. The reaction mixture was diluted by adding 20 mL of distilled water, then extracted with *n*-hexane (3 × 15 mL) to give a corresponding FAME, which was identified by GCMS. The aqueous layer was evaporated to dryness and subjected to Sephadex LH-20 using CHCl_3_:MeOH (10:90) to give LCB and sugar. The base was analyzed by EIMS [22].

## 4. Conclusions

In conclusion, the investigation of the Red Sea strain of the marine cyanobacterium *Moorea producens* led to the isolation of a new cerebroside (**1**) and two new nucleoside derivatives (**2** and **3**), along with two known compounds (**4** and **5**). Their structures were determined using extensive spectroscopic studies. Compounds **1**–**3** showed moderate cytotoxic activity against breast cancer cell lines. 

## Figures and Tables

**Table 1:** NMR spectral data of compound **1** (CDCl_3_, 850 and 213 MHz).

No.	δ_H_ [mult., *J* (Hz)]	δ_C_ (mult.)	HMBC
1	3.91 m, 3.74 m	68.5 CH_2_	2, 3, 1′′
2	3.97 m	59.3 CH	1, 3, 4, 1′
3	3.61 m	73.4 CH	1, 2
4	2.08 m	32.2 CH_2_	2, 5, 6
5	5.35 dt (15.3, 7.6)	129.9 CH	3, 4, 7
6	5.37 dt (15.3, 7.1)	128.8 CH	4, 5, 7
7	2.01 m	32.0 CH_2_	5, 6
8–15	1.27–1.23	30.3–29.0 CH_2_	-
16	1.29 m	22.6 CH_2_	15, 17
17	0.87 t (6.7)	14.1 CH_3_	14, 16
1′	-	173.8 C	-
2′	2.33 t (7.6)	34.4 CH_2_	1′, 4′
3′	1.61 m	24.8 CH_2_	1′, 2′, 4′
4′	1.28 m	28.7 CH_2_	-
5′–17′	1.27–1.23 m	30.3–29.0 CH_2_	-
18′	1.30 m	22.7 CH_2_	17′, 19′
19′	0.89 t (6.8)	14.1 CH_3_	16′, 18′
1′′	4.28 d (7.7)	104.0 CH	1, 2′′, 3′′
2′′	3.65 m	70.2 CH	3′′, 4′′
3′′	3.63 m	71.7 CH	1′′, 2′′, 4′′
4′′	4.02 m	69.5 CH	5′′, 6′′
5′′	3.56 m	74.6 CH	4′′, 6′′
6′′	4.38 m, 4.22 m	62.7 CH_2_	1′′, 5′′
2-N*H*	7.52 d (8.5)	-	2, 3, 1′

**Table 2:** NMR spectral data of compounds **2** and **3** (CD_3_OD, 850 and 213 MHz).

2				3			
No.	δ_H_ [mult., *J* Hz)]	δ_C_ (mult.)	HMBC	No.	δ_H_ [mult., *J* (Hz)]	δ_C_ (mult.)	HMBC
2	-	151.7 C		2	-	151.7 C	-
4	-	162.8 C		4	-	162.8 C	-
5	5.69 d (8.5)	102.6 CH	4, 6	5	5.69 d (8.5)	102.6 CH	6
6	7.98 d (8.5)	142.5 CH	2, 4, 5, 1′	6	7.98 d (8.5)	142.5 CH	2, 4, 5
1′	6.27 t (6.8)	86.6 CH	2, 6, 2′	1′	6.26 t (6.8)	86.2 CH	2′, 3′
2′	2.28 m	41.4 CH_2_	1′, 3′	2′	2.21 m	41.2 CH_2_	1′, 3′
3′	4.38 m	72.2 CH		3′	4.36 m	72.3 CH	
4′	3.89 m	89.0 CH		4′	3.91 m	88.8 CH	
5′	3.75 m, 3.71 m	62.8 CH_2_		5′	3.75 m, 3.71 m	62.8 CH_2_	
1′′	-	183.4 C		1′′	3.16 t (7.6)	42.0 CH_2_	2, 4, 2′′, 3′′
2′′	1.95 s	23.5 CH_3_	1′′	2′′	2.95 t (7.6)	34.7 CH_2_	3′′, 4′′, 8′′
				3′′	-	137.9 C	-
				4′′, 8′′	7.27 brd (6.8)	129.8 CH	2′′, 6′′
				5′′, 7′′	7.35 m	130.0 CH	3′′, 4′′, 8′′
				6′′	7.27 m	128.3 CH	

**Table 3:** Cytotoxic activities of compounds **1**–**5**.

Compound	IC_50_ (μg/mL)		
	Colorectal Carcinoma (HCT-116)	Hepatocellular Carcinoma (HepG2)	Breast Cancer (MCF-7)
**1**	>50	>50	20.5
**2**	>50	>50	18.2
**3**	>50	>50	22.8
**4**	NT	NT	NT
**5**	NT	NT	NT
**Doxorubicin**	0.789	0.621	0.415

NT = Not tested.

## Supplementary Materials

The following are available online at http://www.mdpi.com/1420-3049/21/3/324/s1, Figure S1: 1D and 2D NMR spectra of compounds **1**–**3**.
