# Supplementary material for: New Cerebroside and Nucleoside Derivatives from a Red Sea Strain of the Marine Cyanobacterium Moorea producens
Source: Molecules. 2016 Mar 9;21(3):324. doi: 10.3390/molecules21030324 (PMC6272925; doi:10.3390/molecules21030324)
Supplement: Supplementary file 1 [file molecules-21-00324-s001.pdf]

Supplementary Materials: New Cerebroside and Nucleoside Derivatives from a Red Sea Strain of the Marine Cyanobacterium *Moorea producens*

Diaa T.A. Youssef, Sabrin R.M. Ibrahim, Lamiaa A. Shaala, Gamal A. Mohamed and Zainy M. Banjar

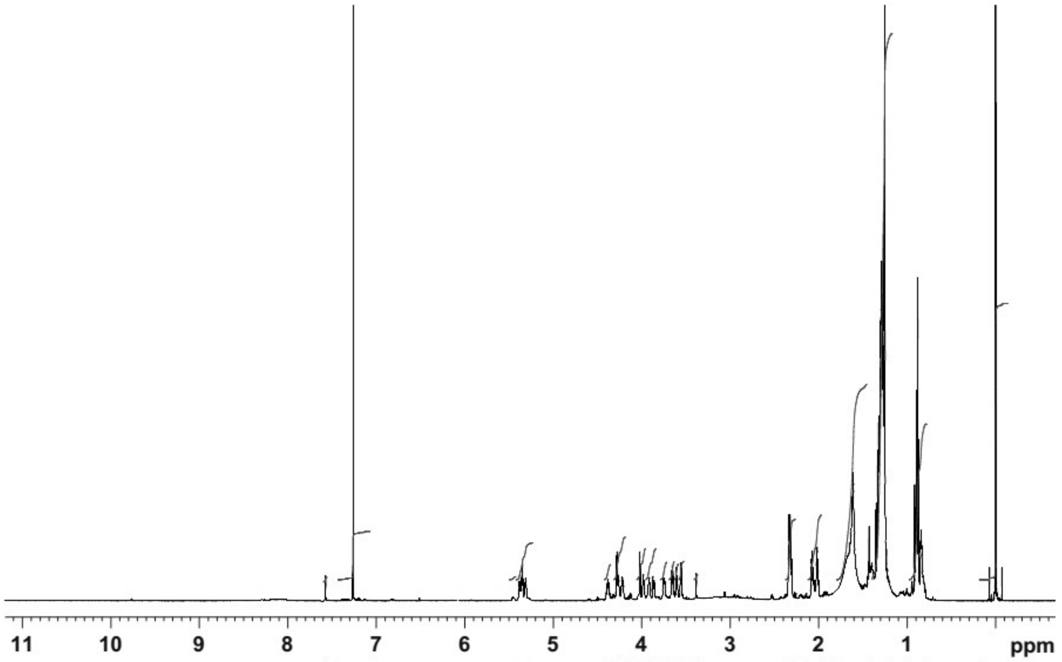

Figure S1. ^{1}H-NMR spectrum of compound **1** (850 MHz, $CDCl_3$).

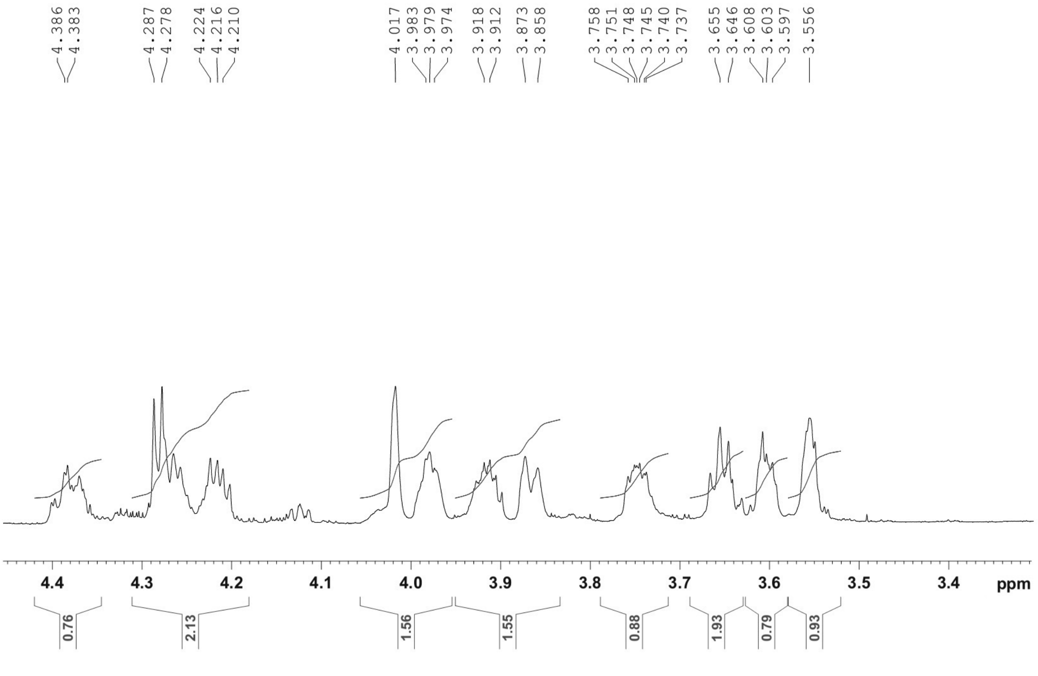

Figure S2. Partial ^{1}H-NMR spectrum of compound **1** (850 MHz, $CDCl_3$).

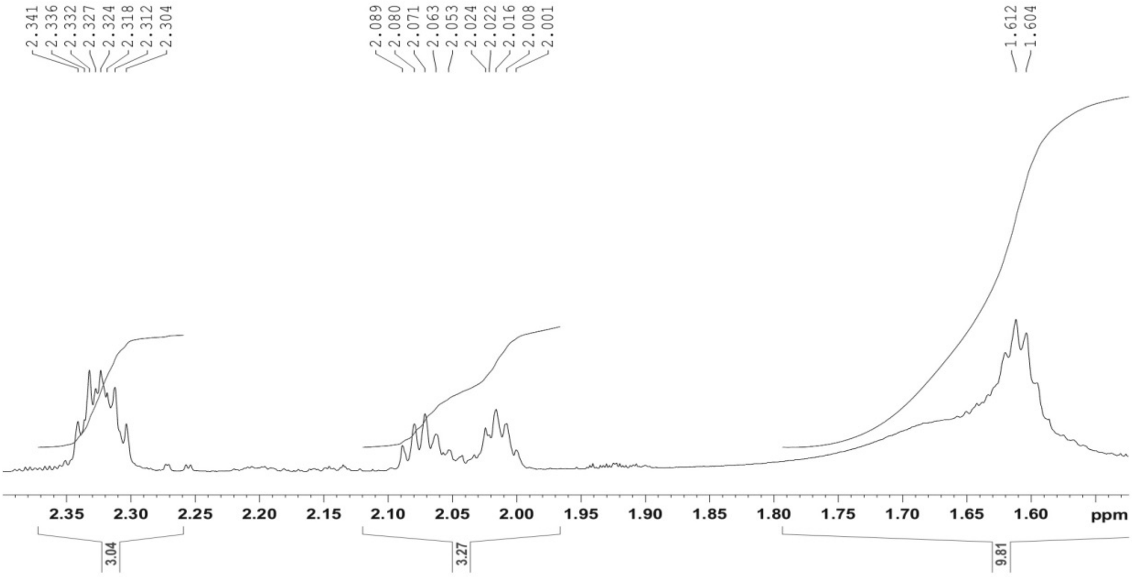

Figure S3. Partial ^{1}H-NMR spectrum of compound **1** (850 MHz, $CDCl_3$).

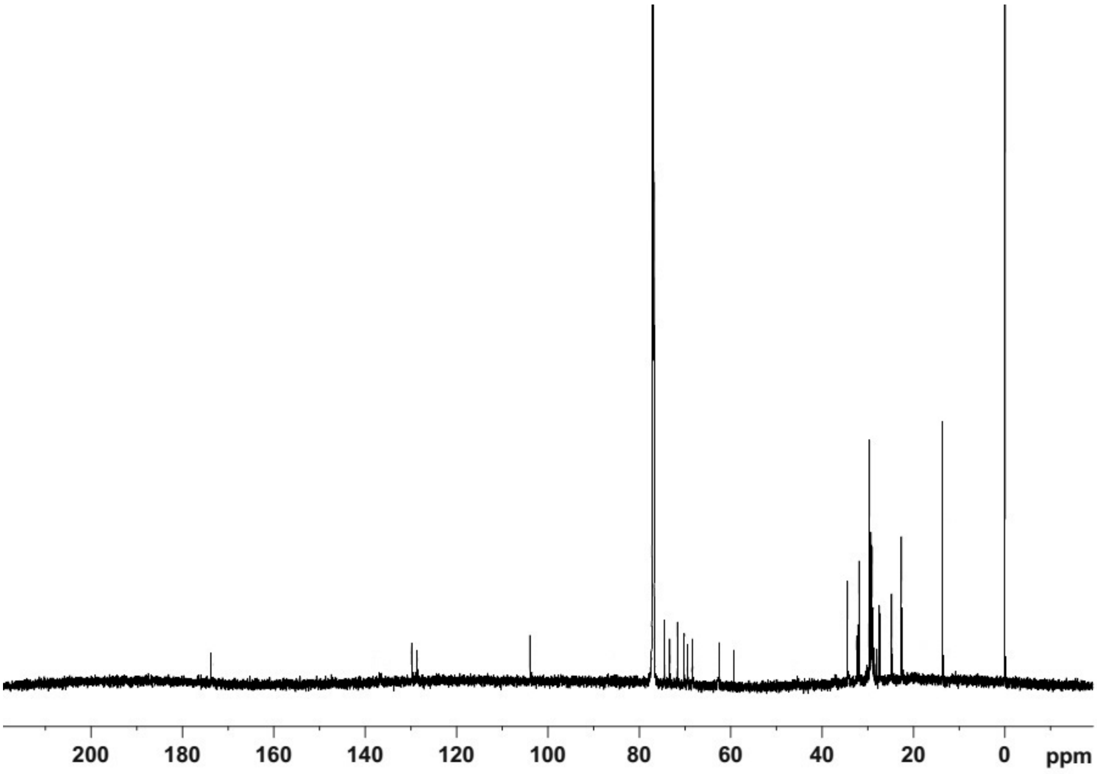

Figure S4. ^{13}C-NMR spectrum of compound **1** (213.8 MHz, $CDCl_3$).

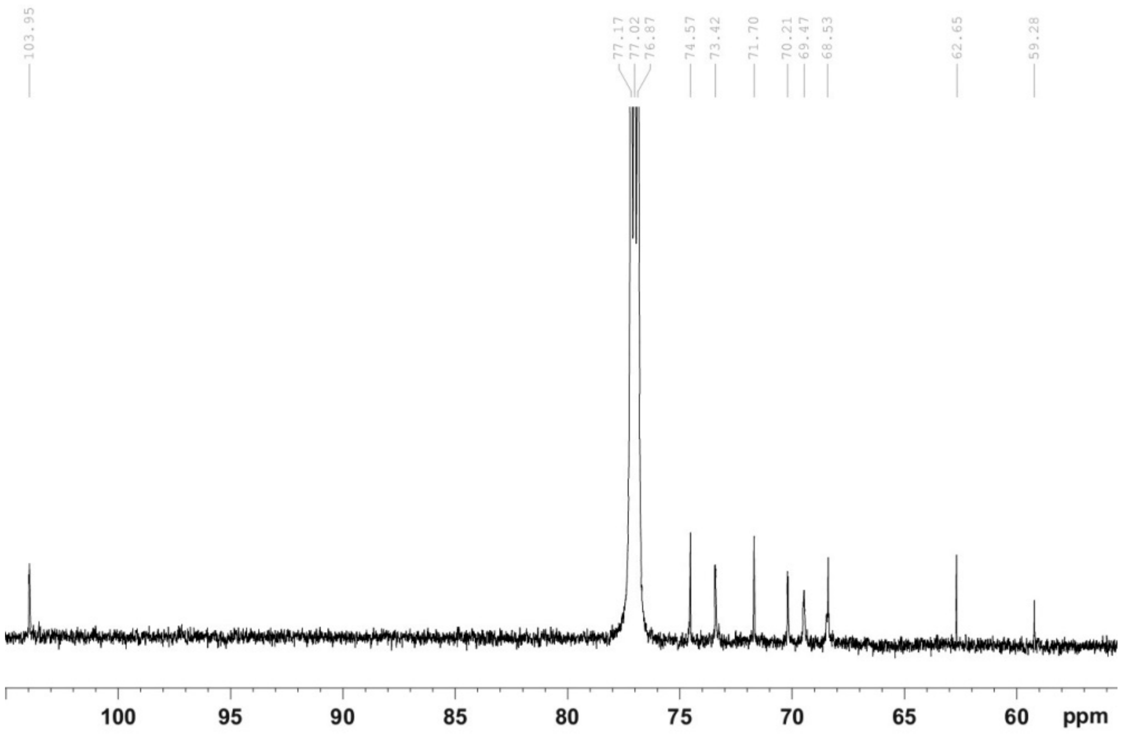

Figure S5. Partial ^{13}C-NMR spectrum of compound **1** (213.8 MHz, $CDCl_3$).

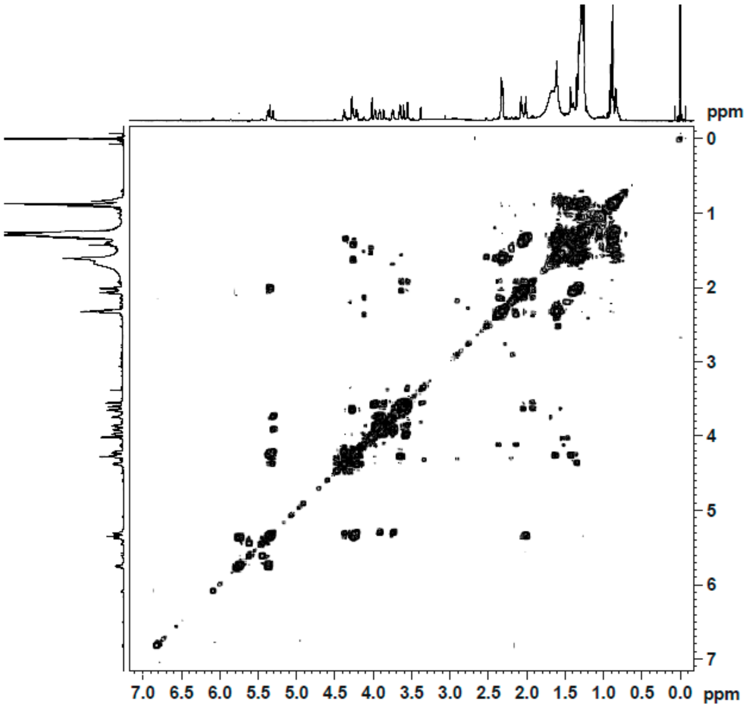

Figure S6. COSY spectrum of compound **1**.

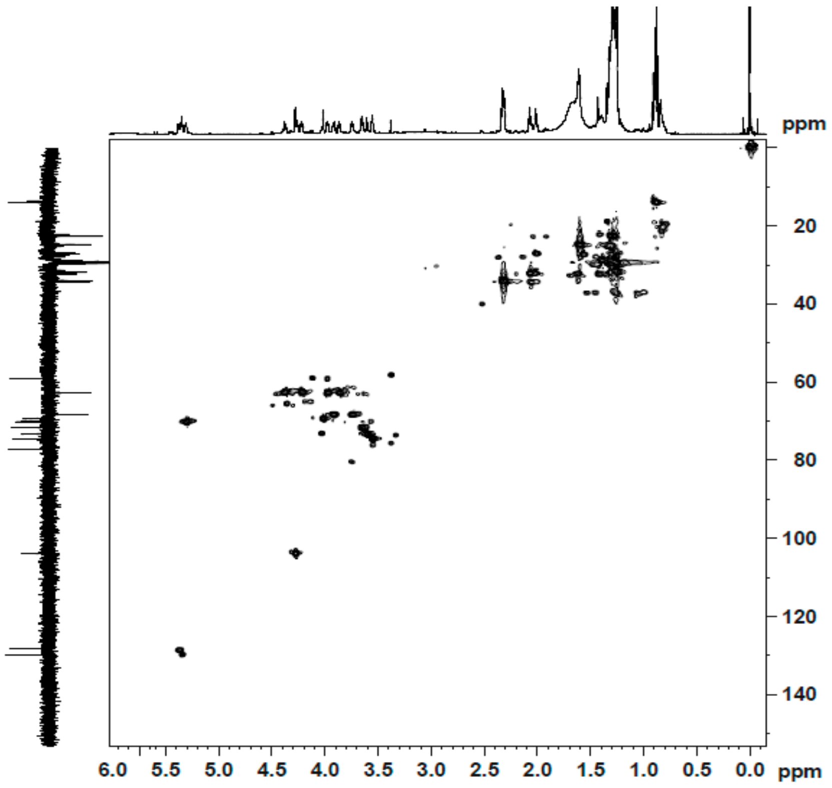

Figure S7. HSQC spectrum of compound **1**.

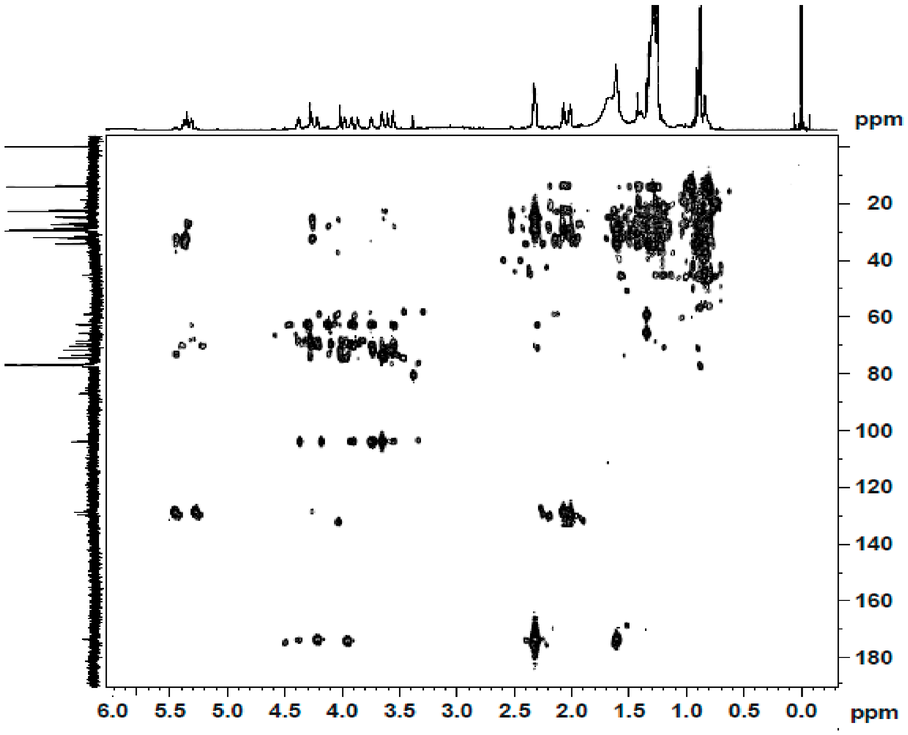

Figure S8. HMBC spectrum of compound **1**.

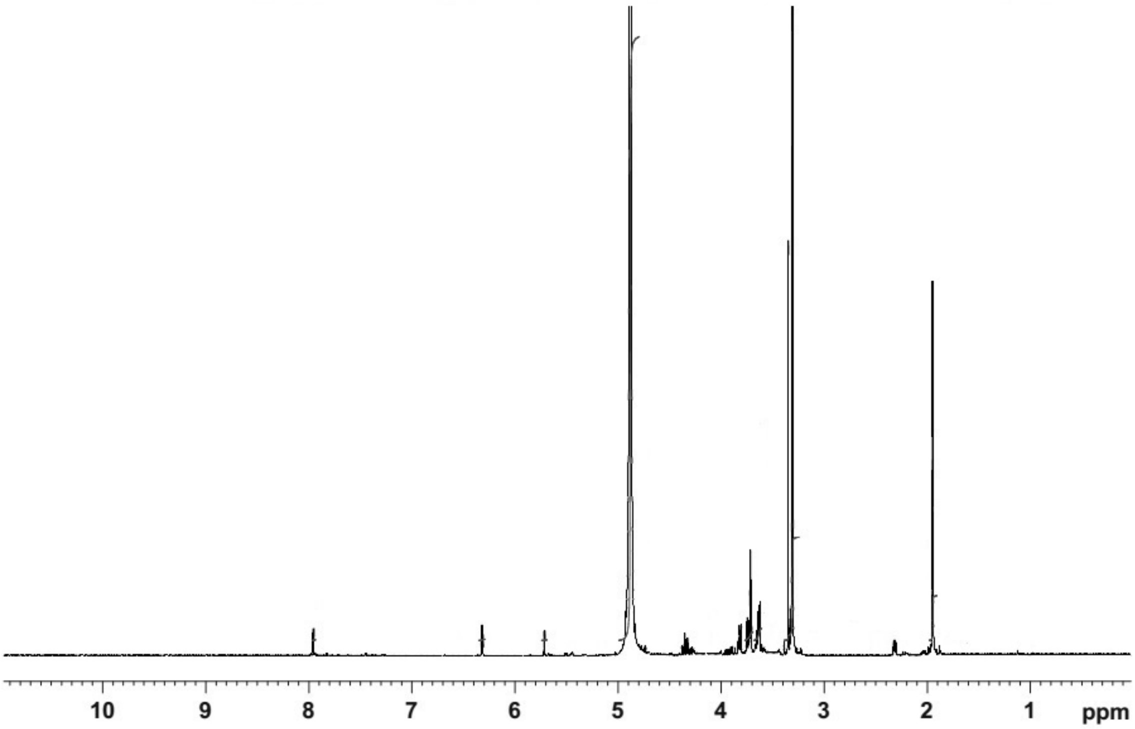

Figure S9. ^{1}H-NMR spectrum of compound **2** (850 MHz, CD_3OD).

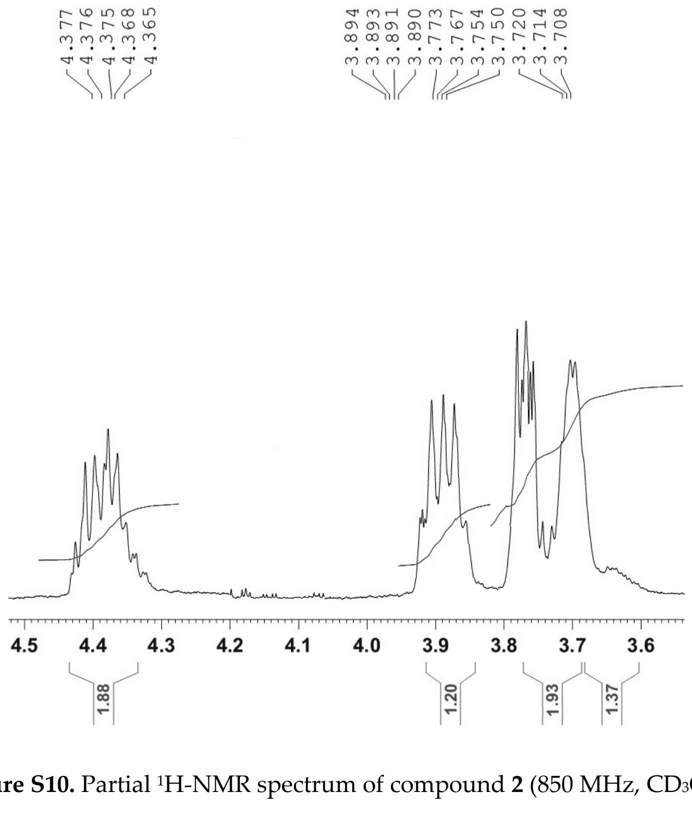

Figure S10. Partial ^{1}H-NMR spectrum of compound **2** (850 MHz, CD_3OD).

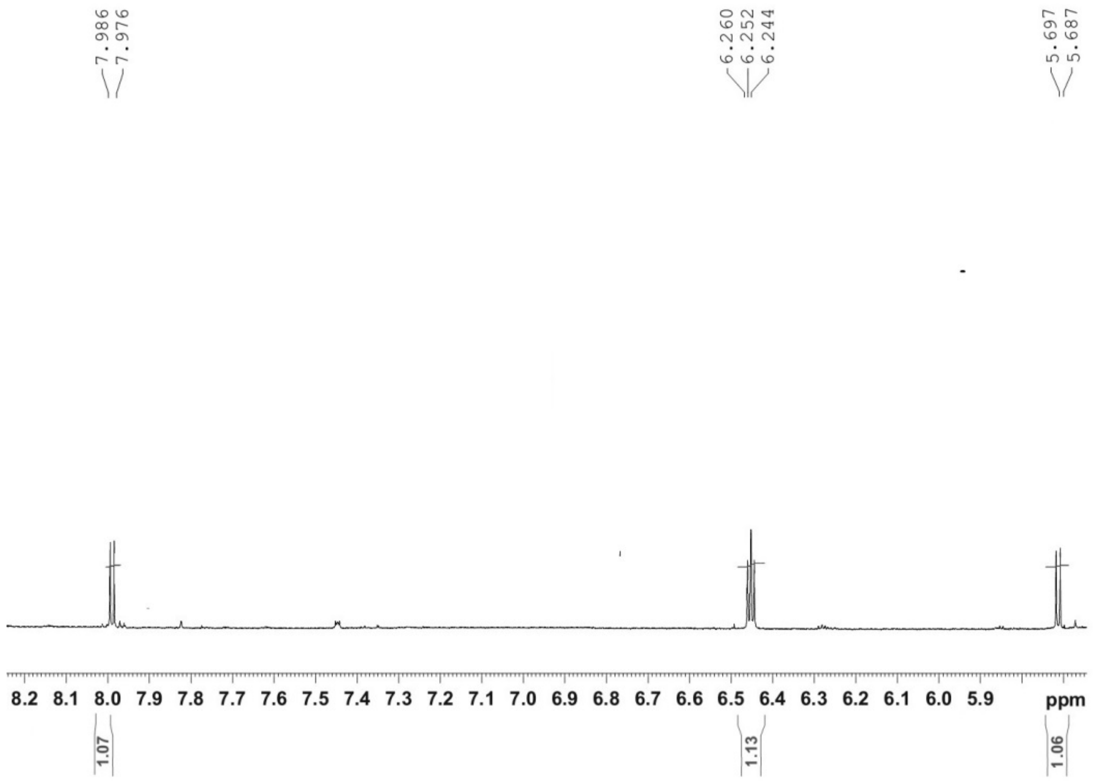

Figure S11. Partial ^{1}H-NMR spectrum of compound **2** (850 MHz, CD_3OD).

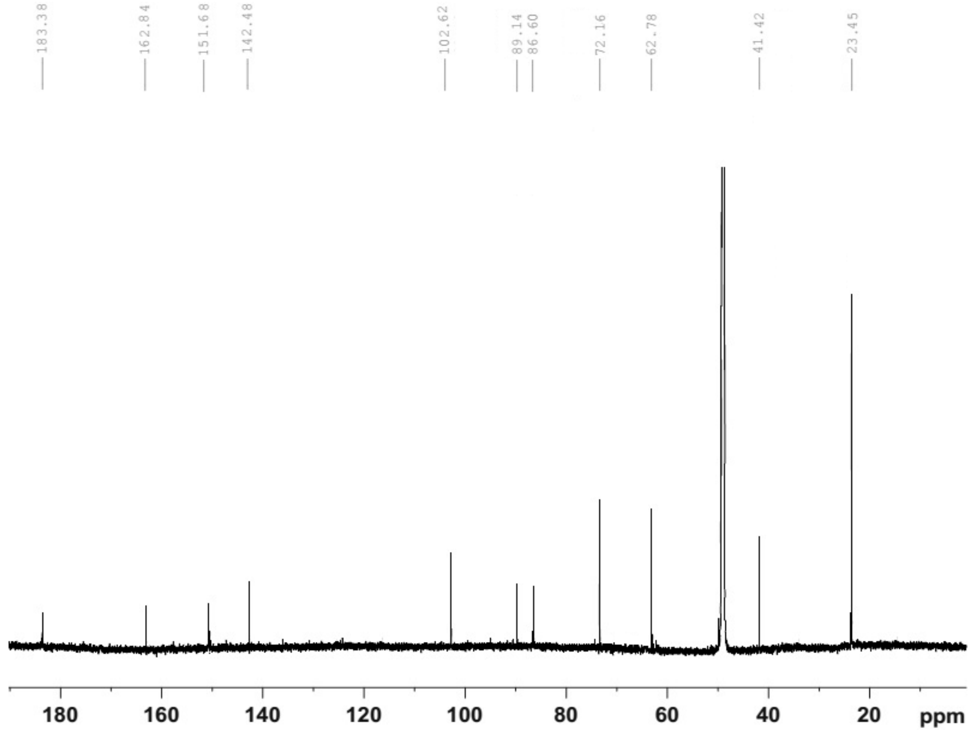

Figure S12. ^{13}C-NMR spectrum of compound **2** (213.8 MHz, CD_3OD).

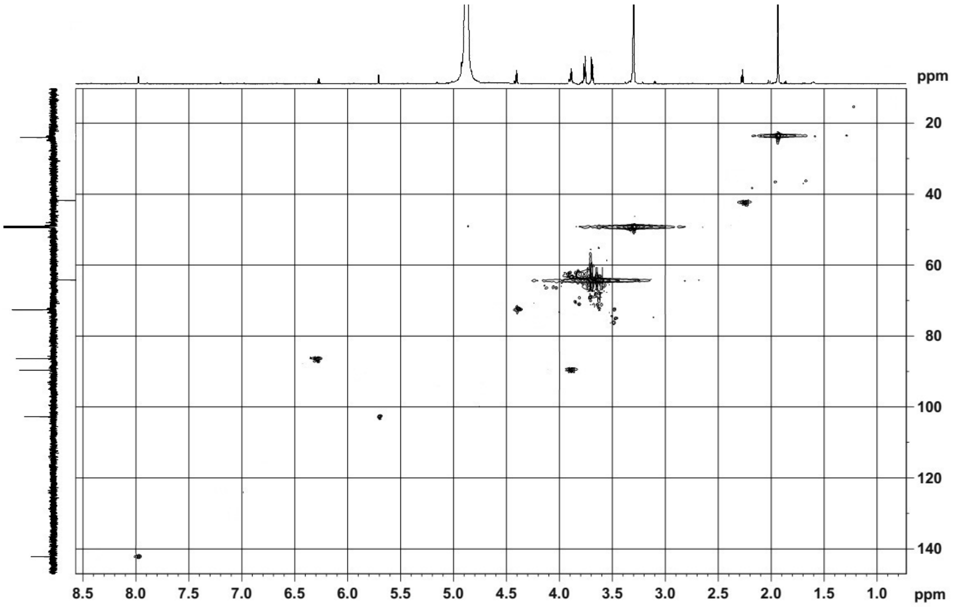

Figure S13. HSQC spectrum of compound **2**.

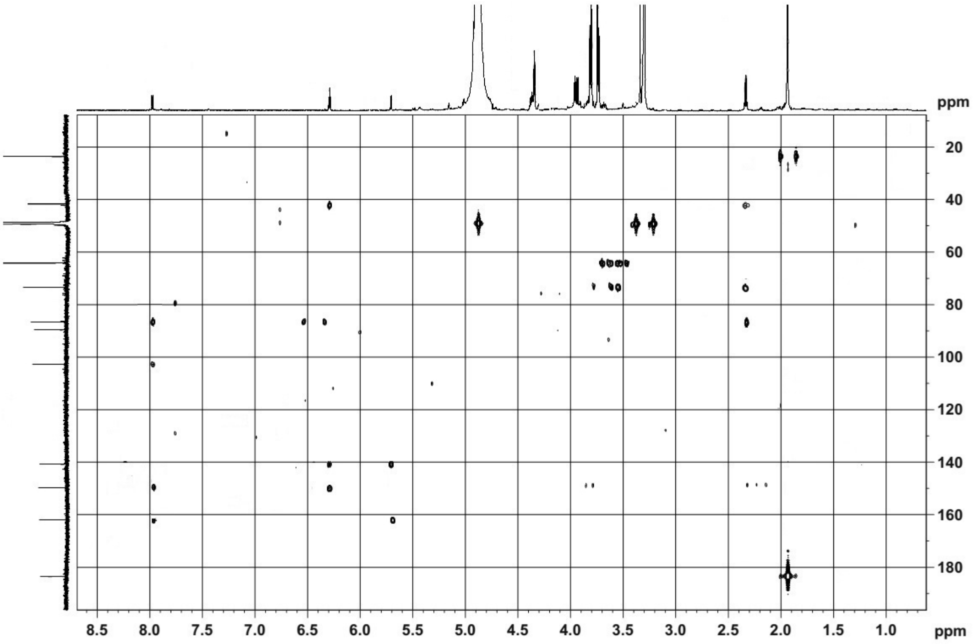

Figure S14. HMBC spectrum of compound **2**.

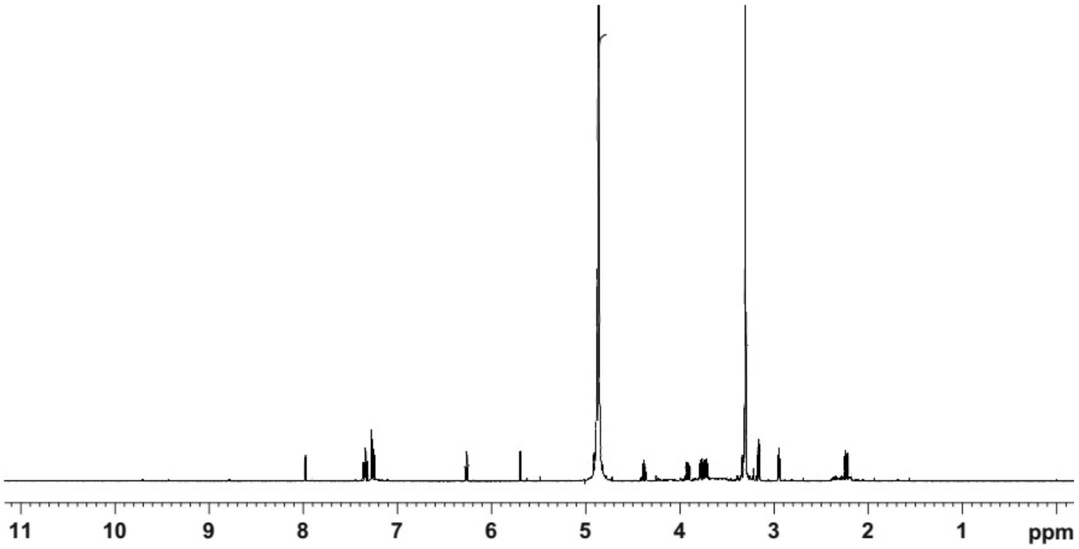

Figure S15. ^{1}H-NMR spectrum of compound **3** (850 MHz, CD_3OD).

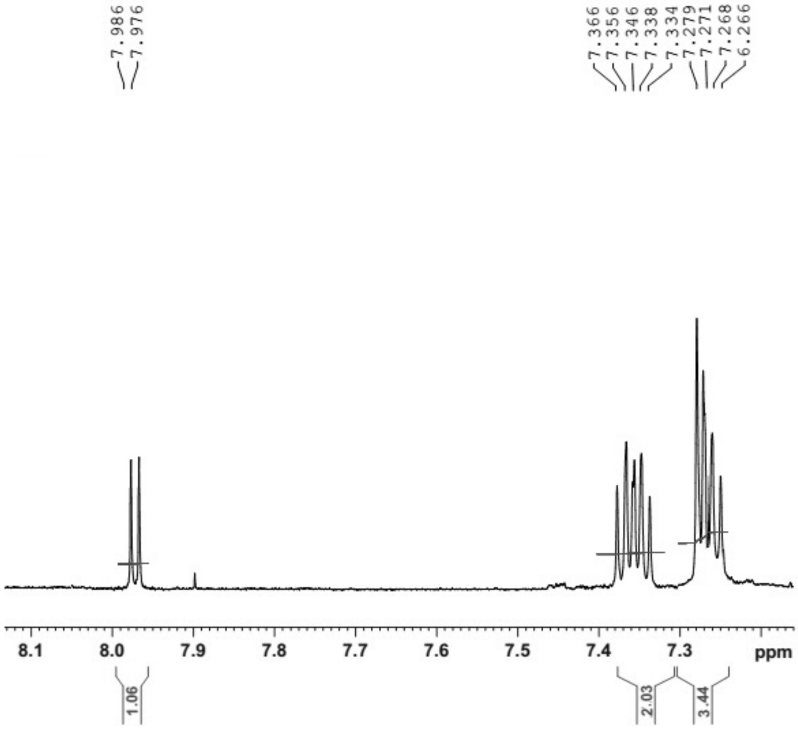

Figure S16. Partial ^{1}H-NMR spectrum of compound **3** (850 MHz, CD_3OD).

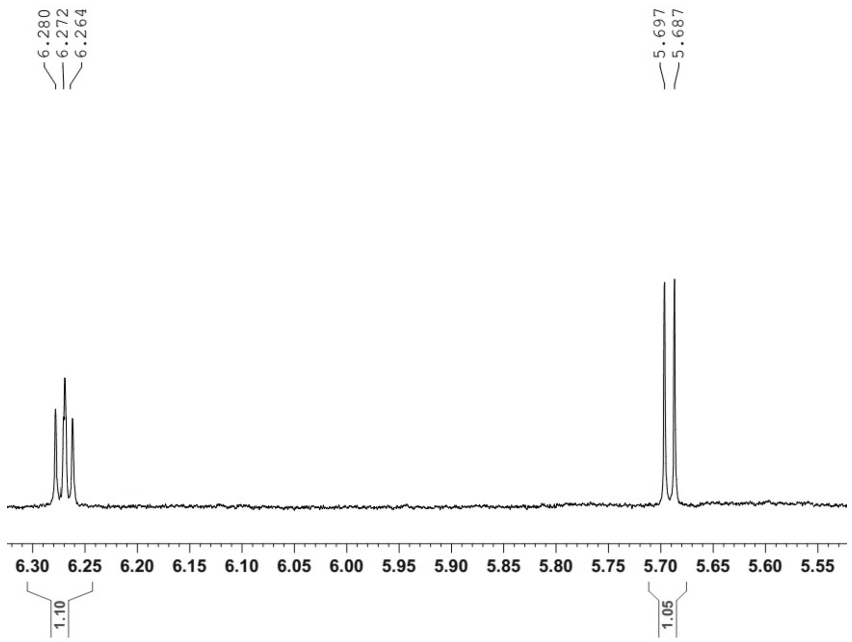

Figure S17. Partial ^{1}H-NMR spectrum of compound **3** (850 MHz, CD_3OD).

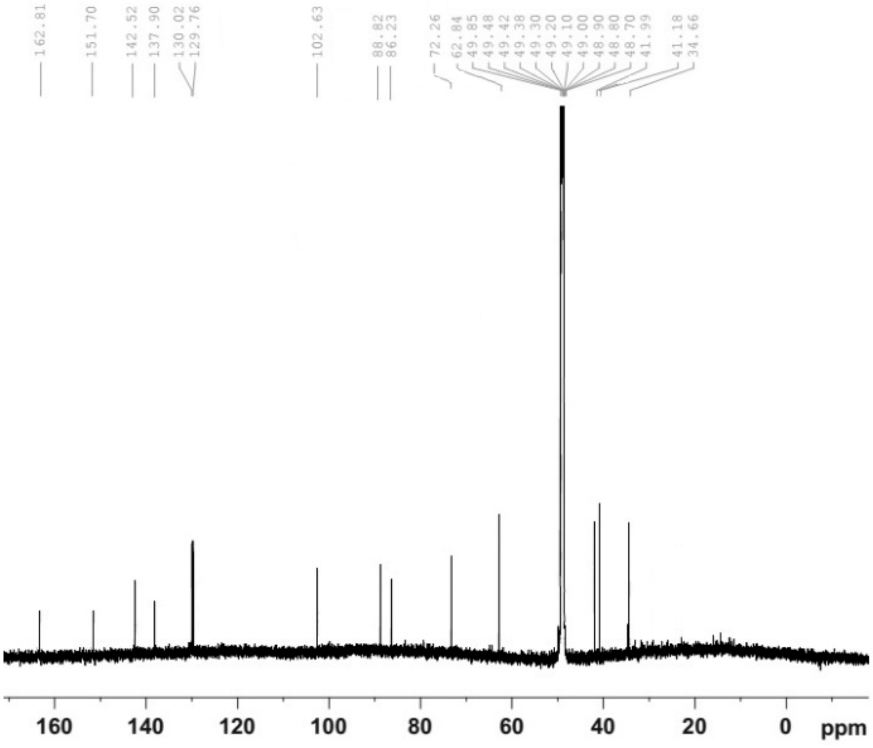

Figure S18. ^{13}C-NMR spectrum of compound **3** (213.8 MHz, CD_3OD).

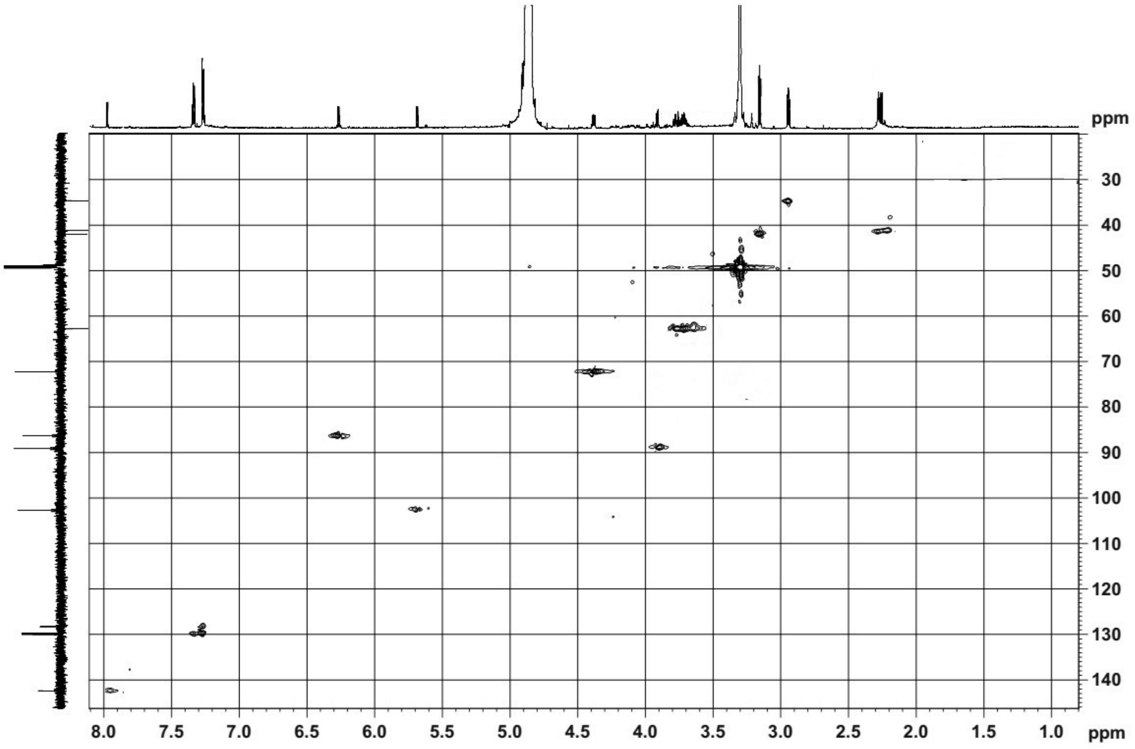

Figure S19. HSQC spectrum of compound **3**.

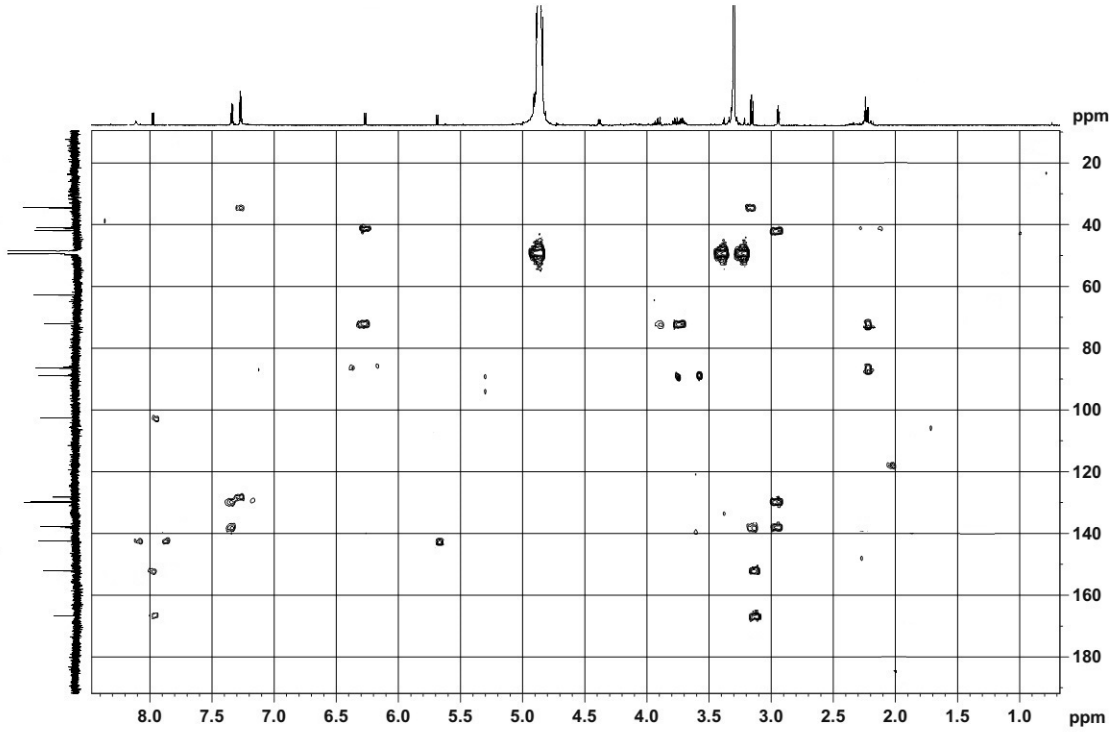

Figure S20. HMBC spectrum of compound **3**.
